# Acute respiratory responses to the use of e-cigarette: an intervention study

**DOI:** 10.1038/s41598-019-43324-1

**Published:** 2019-05-02

**Authors:** Grzegorz M. Brożek, Mateusz Jankowski, Jan E. Zejda

**Affiliations:** Department of Epidemiology, School of Medicine, Medical University of Silesia in Katowice, Medykow 18 Str, 40-752 Katowice, Poland

**Keywords:** Epidemiology, Epidemiology

## Abstract

The goal of our study was to assess acute respiratory responses to using e-cigarettes in exclusive e-cigarette users (E-Group) and dual users (T/E-Group) and to compare these effects with responses to smoking tobacco-cigarettes in tobacco smokers (T-Group). The study included 120 adults (age: 21.7 ± 2.1 years) divided into 4 groups (n = 30 each): Controls, T-, E-, T/E-Group. Spirometric status, O_2_ saturation, exhaled FeNO levels, exhaled CO levels, and airway temperature were assessed before the use of an e-cigarette (E-, T/E-Group) or tobacco cigarette as well as ‘minute 1’ and ‘minute 30’ after smoking. Controls used an e-cigarette without e-liquid. Lower (p < 0.05) baseline values of FeNO were found in T-Group (15.4 ppb) and in T/E-Group (15.0 ppb) than in Controls (19.6 ppb). Following exposure, and compared with Controls, T-, and T/E-Group had a significant decrease (p < 0.05) in PEF and MEF75. Mean FeNO values decreased on ‘minute 1’ in T-Group (by 2.1 ppb), E-Group (by 1.5 ppb) and in T/E-Group (by 2.2 ppb). Other effects included increase in temperature of exhaled air (p < 0.05). The use of e-cigarettes is associated with decreased FeNO and airflow indices (PEF, MEF75), but an increase in airway temperature. These changes are similar to those after exposure to tobacco cigarette smoke.

## Introduction

The electronic cigarette (e-cigarette) is an electronic nicotine delivery system (ENDS), which is one of alternatives to traditional tobacco cigarettes^1^. The prevalence of e-cigarette use differs across populations in Europe^2^. The highest percentage of e-smokers is observed among young adults aged 15–24 years, while the dominant group of e-cigarette users are dual users, using both e-cigarettes and smoking traditional cigarettes^2–4^.

During the use of e-cigarettes, a special inhalation solution called “e-liquid“ is aerosolized via heating. E-liquids differ in terms of chemical composition, flavors and nicotine content^5,6^. There are several harmful products inhaled by e-smokers including acroleine, acetone, acetaldehyde, formaldehyde, propanal, nicotine, o-methyl-benzaldehyde and carcinogenic nitrosamines^5,7,8^.

The health effects of using e-cigarettes are under investigation. Because e-cigarettes have only been on the market for a short time, the results from long-term observational studies are not available. The current knowledge regarding potential health consequences of e-cigarette use is based on experimental animal studies or short-term studies, assessing the acute effects of exposure to the aerosol generated during e-cigarette use^9–15^. Furthermore currently available reports differ in the principal findings^10,11^.

The goal of our study was to assess acute, short term respiratory responses (airflow, FeNO, O_2_ saturation, exhaled air temperature) to using an e-cigarette in exclusive e-smokers and dual users and to compare these effects with responses to smoking a tobacco-cigarette in exclusive tobacco smokers.

## Material and Methods

### Subjects

This study is a continuation of a questionnaire survey conducted as part of the multicenter, international project YoUng People E-Smoking Study (YUPESS)^16^. We surveyed 3002 participants and identified 39 exclusive e-smokers and 54 dual users from the center in Katowice, Poland. All e-cigarette users (n = 93) were invited to participate in the current part of the study. From these, 26 refused and 7 met exclusion criteria. Exclusion criteria included the occurrence of any chronic diseases, history of lung conditions (eg.: asthma or bronchial hyperactivity in childhood), presence of any allergic diseases, medication intake within the last 2 weeks, acute illnesses or infections in the last 2 weeks, influenza vaccination in the last 2 weeks, or current pregnancy or lactation.

After all 60 e-cigarette users were recruited (30 exclusive e-cigarette users and 30 dual users), 30 people were drawn randomly from a group of cigarette smokers (n = 433) and non-smokers (control group, n = 2476). Participants who took part in the full cross-sectional study compared to the group involved in this sub-study did not differ (p > 0.05) by age or sex.

Finally, 120 healthy adults (aged 21.7 ± 2.1 y-old) were enrolled. Subjects were divided into 4 groups (n = 30 each) based on their self-declared smoking status: T-Group composed of T-cigarette smokers (T-Subjects), E-Group composed of E-Cigarette users (E-Subjects), T/E-Group composed of dual users (T/E-Subjects) and control group (C-Group) composed of C-Subjects.

All subjects were asked to avoid tobacco cigarette or e-cigarette smoke on the examination day (abstinence for a minimum of 6 hours before the test) and not to eat or drink for at least 2 hours before the examination.

### Study design

This was a laboratory-based intervention study (pre-post-post) performed at the Respiratory Function Laboratory at the Department of Epidemiology, Medical University of Silesia in Katowice. Regular e-cigarette users were asked to use their own e-cigarette device. In every case, e-cigarettes were filled with multi-fruit flavoured e-liquid containing 12 mg/ml of nicotine, which was randomly selected from commonly available e-liquids. Physical examination and a short interview were performed to assess the current health status of each participant. The participants were informed about the measurement techniques to be used in the study including: (1) O_2_ saturation; (2) concentration of nitric oxide (FeNO) in exhaled air; (3) exhaled carbon monoxide (CO); (4) temperature of exhaled air; (5) spirometric testing. All measurements were performed in the order specified above.

E-cigarette users (E-Group) and dual users (T/E-Group) were instructed to use e-cigarettes in accordance with everyday habits for 5 minutes. Cigarette smokers (T-Subjects) were asked to smoke a cigarette of one of the popular brands (0.6 mg nicotine per one cigarette) according to their everyday habits. The control subjects were asked to simulate the use of e-cigarettes (a device without e-liquid where aerosol was not created or inhaled).

Before exposure (e-cigarette, conventional cigarette, empty device), baseline O_2_ saturation, exhaled FeNO and CO levels, exhaled air temperature, and spirometric tests were completed. The measurements were repeated immediately (1 minute) following exposure and 30 minutes following exposure. In the control group, the post-measurements were obtained only in the first minute after the simulated exposure. According to the decision of the Ethics Committee, the third measurement (after 30 min) was not allowed in the control group when the first and the second measurement results did not differ.

Informed written consent was obtained from all individual participants included in the study.

All procedures performed in studies involving human participants were in accordance with the ethical standards of the institutional and/or national research committee and with the 1964 Helsinki declaration and its later amendments or comparable ethical standards. The study protocol was approved by the Ethics Committee of the Medical University of Silesia in Katowice (decision number: KNW/0022/KB1/37/I/17).The study was funded by an internal research University grant KNW-1-024/K/7/0.

### Lung functions assessment

#### Spirometry

Airflow and lung volume were measured in the sitting position with a nose clip according to the ATS/ERS guidelines^17,18^. The measurements were made using the EasyOne 2001 spirometer and included FEV1 (forced expiratory volume in 1 second), FVC (forced vital capacity), FEV1% FVC (forced expiratory volume in one second to forced vital capacity), PEF (peak expiratory flow), and MEF75,50,25 (maximal expiratory flow at 75%, 50% and 25% of FVC). Expiratory maneuvers were repeated until a minimum of three technically correct, repeatable measurements were obtained.

#### O_2_ saturation

The O_2_ measurement was made in the sitting position using the PULSOX 2 electronic pulse meter. The result was recorded 15 seconds after the start of the measurement.

#### Exhaled nitric oxide (FeNO)

FeNO measurements were made using the standard procedure in the sitting position with a clip attached to the nose using a NIOX MINO device for measuring fractional exhaled nitric oxide (FeNO)^19^. Subjects were instructed to breathe as deeply as possible through the mouthpiece to achieve maximum lung filling and then to exhale 50 ml/sec for 10 s. The measured FeNO values were expressed in ppb (parts per billion).

#### Exhaled carbon monoxide (CO)

During the measurement, requirements regarding the length and intensity of the exhalation were taken into account. The measurements were made using the PiCo Smokylyzer device. The measured CO levels were expressed in ppm (parts per million).

#### Exhaled breath temperature

The measurement was made using the standard procedure in the sitting position using an X-Halo Breath Thermometer^20^. Respondents were instructed to breathe according to the procedure recommended by the device manufacturer. The temperature of exhaled air was expressed in Celsius degrees (°C).

### Statistical analysis

Data analysis was performed using Statistica 12. Data were described using means, standard deviations, and medians for quantitative variables and percentages for qualitative variables. Normality of distributions was tested using the Shapiro-Wilk test. Differences in the distribution of quantitative variables were evaluated based on the results of the Student’s t-test or non-parametric tests (U Mann-Whitney), and in the case of repeated variables the paired Student-t-test and Wilcoxon test were used. Comparisons involving more than two groups for quantitative variables were completed using the analysis of variance (ANOVA) and its nonparametric equivalents for independent comparisons while Friedman’s ANOVA was used for repeated variables. The post-hoc Dunn’s test was used to control for multiple comparisons. Differences in the distribution of categorical variables were evaluated based on chi-square test results.

Changes in spirometric status, FeNO levels, O_2_ saturation, exhaled CO levels and temperature of exhaled air were assessed by calculating relative differences (in %) according to the formula: (Baseline Value − Post-exposure Value)/Baseline Value. Relative differences were calculated separately for minute 1 vs baseline measurements and for minute 30 versus baseline measurements.

In relation to relative differences, the results of simple analyses were verified by multivariate linear regression models. The effect of smoking category (T-Group vs E-Group, E-Group vs T/E-Group, T-Group vs T/E-Group) on relative differences in the examined indices was assessed using a general linear regression model allowing adjustment for age, sex and height.

Statistical significance was assessed using an alpha of 0.05 and interpretation of the results of multivariate analyses was based on the criterion p < 0.1.

## Results

The descriptive characteristics of the four groups are presented in Table 1. The groups did not differ significantly in terms of the examined variables.

**Table 1:** Description of the study groups (sex, age, height and weight).

	Overall n = 120	C-Group n = 30	T-Group n = 30	E-Group n = 30	T/E-Group n = 30	p
Male [%]	59.2	50.0	50.0	63.3	73.3	0.2*
Female [%]	40.8	50.0	50.0	36.7	26.7	
Age, mean [years ± SD]	22.6 ± 2.2	22.9 ± 1.9	23.2 ± 1.6	22.2 ± 2.3	22.3 ± 2.7	0.1**
Height, mean [cm ± SD]	174.2 ± 8.8	174.5 ± 9.2	172.5 ± 7.9	175.5 ± 9.4	174.6 ± 8.8	0.5**
Weight, mean [kg ± SD]	71.3 ± 15.0	69.2 ± 14.1	67.1 ± 14.5	74.7 ± 14.4	75.4 ± 16.3	0.06**
Cigarette smoking duration, months [mean ± SD]	—	—	50.0 ± 32.0	—	67.3 ± 30.5	0.03***
Cigarettes per day [mean ± SD]	—	—	6.2 ± 4.5	—	8.0 ± 5.9	0.6***
E-cigarette using duration, months [mean ± SD]	—	—	—	29.0 ± 24.1	27.7 ± 17.4	0.6***
E-cigarette sessions per day [mean ± SD]	—	—	—	15.6 ± 13.8	14.7 ± 11.9	0.9***

Legend: SD - standard deviation; *result of Chi-square test; **result of ANOVA; ***result of Mann-Whitney U test.

### Baseline values

All baseline individual measurements were within the range of reference values and are presented in Tables 2 and 3. The study groups differed significantly only in terms of FeNO levels (p = 0.02) and exhaled CO concentration (p = 0.0001). Compared with the control group, lower values of FeNO were found in T-Group (p = 0.01) and in T/E- Group (dual users) (p = 0.006). CO concentrations were significantly lower in the control group than in T-Group (p = 0.003), E-Group (p = 0.01) and T/E-Group (p = 0.0001). Subjects did not differ in terms of baseline values of heart rate and blood pressure (p > 0.05).

**Table 2:** Changes in O_2_ saturation, FeNO and CO levels and in exhaled air temperature after exposure to t-cigarette, e-cigarette or simulated exposure.

	Baseline mean ± SD (min-max)	Minute 1 mean ± SD (min-max)	Minute 30 mean ± SD (min-max)	p_I-II-III*_	p_I-II**_	p_II-III**_	p_I-III**_
**O**_**2**_ **Saturation [%]**							
C-Group n = 30	98.23 ± 0.97 (97–100)	98.00 ± 1.08 (96–100)	—	—	0.1^***^	—	—
T-Group n = 30	97.97 ± 1.07 (96–100)	97.40 ± 1.13 (95–99)	98.00 ± 1.14 (95–100)	0.009	0.01	0.01	0.9
E-Group n = 30	97.63 ± 1.09 (96–100)	97.70 ± 1.26 (95–100)	97.70 ± 1.06 (95–100)	0.8	0.8	0.9	0.7
T/E-Group n = 30	97.73 ± 0.94 (96–100)	97.53 ± 1.01 (96–100)	97.60 ± 0.93 (96–100)	0.5	0.2	0.8	0.4
**FeNO [ppb]**							
C-Group n = 30	19.63 ± 6.17 (11–33)	19.46 ± 5.85 (7–30)	—	—	0.7***	—	—
T-Group n = 30	15.43 ± 5.57 (6–28)	13.30 ± 4.91 (6–25)	15.90 ± 5.83 (8–30)	0.0001	0.0001	0.0001	0.2
E-Group n = 30	17.43 ± 8.93 (5–39)	15.93 ± 8.24 (5–35)	18.4 ± 9.07 (6–39)	0.0001	0.0001	0.0001	0.02
T/E-Group n = 30	15.06 ± 8.44 (5–37)	12.86 ± 7.22 (5–31)	15.46 ± 8.19 (6–36)	0.0001	0.0001	0.0001	0.5
**Exhaled CO level [ppm]**							
C-Group n = 30	1.90 ± 0.66 (1–3)	2.03 ± 0.67 (1–3)	—	—	0.2***	—	—
T-Group n = 30	4.10 ± 4.28 (1–22)	7.77 ± 4.26 (3–24)	6.77 ± 3.90 (2–22)	0.0001	0.0001	0.0002	0.0001
E-Group n = 30	2.43 ± 0.82 (1–4)	2.63 ± 0.81 (1–4)	2.60 ± 0.90 (1–4)	0.1	0.09	0.8	0.2
T/E-Group n = 30	5.77 ± 4.15 (2–17)	5.63 ± 3.79 (2–16)	4.80 ± 3.09 (1–13)	0.0001	0.4	0.0005	0.001
**Exhaled air temperature [°C]**							
C-Group n = 30	34.28 ± 0.47 (33.41–35.29)	34.34 ± 0.50 (33.04–35.57)	—	—	0.4***	—	—
T-Group n = 30	34.28 ± 0.43 (33.65–35.09)	34.27 ± 0.55 (33.03–35.43)	34.58 ± 0.39 (33.76–35.40)	0.0001	0.7	0.0003	0.0001
E-Group n = 30	34.33 ± 0.61 (32.73–35.19)	34.48 ± 0.48 (33.00–35.19)	34.55 ± 0.40 (33.62–35.23)	0.03	0.04	0.4	0.03
T/E-Group n = 30	34.39 ± 0.4 (33.62–35.02)	34.56 ± 0.35 (33.38–35.09)	34.60 ± 0.29 (33.89–35.12)	0.02	0.004	0.4	0.003

Legend: SD - standard deviation; min-max - minimum and maximum value range; I, II, III - order of measurements; *result of Friedman ANOVA; **Post-hoc Dunn’s test; ***Wilcoxon signed-ranked test.

**Table 3:** Changes in spirometric variables after exposure to t-cigarette, e-cigarette or simulated exposure.

	Baseline mean ± SD (min-max)	Minute 1 mean ± SD (min-max)	Minute 30 mean ± SD (min-max)	p_I-II-III*_	p_I-II**_	p_II-III**_	p_I-III**_
**Control Subjects n** = **28**							
FVC [l]	4.85 ± 1.02	4.90 ± 1.04	—	—	0.1^***^	—	—
FVC [%]	104.60 ± 10.24	105.50 ± 10.28	—	—	0.2^***^	—	—
FEV_1_[l]	4.05 ± 0.75	4.07 ± 0.78	—	—	0.8^***^	—	—
FEV_1_[%]	102.32 ± 9.35	102.68 ± 10.00	—	—	0.9^***^	—	—
FEV_1_/FVC [%]	84.28 ± 6.94	83.71 ± 7.08	—	—	0.06^***^	—	—
PEF [l/s]	7.98 ± 1.94	7.74 ± 1.86	—	—	0.09^***^	—	—
PEF [%]	90.68 ± 12.38	87.90 ± 12.25	—	—	0.09^***^	—	—
MEF_25_ [l/s]	2.30 ± 0.69	2.22 ± 0.74	—	—	0.07^***^	—	—
MEF_25_ [%]	93.57 ± 28.31	90.29 ± 30.30	—	—	0.08^***^	—	—
MEF_25–75_ [l/s]	4.31 ± 1.06	4.27 ± 1.10	—	—	0.1^***^	—	—
MEF_25–75_ [%]	92.75 ± 18.21	92.57 ± 19.13	—	—	0.2^***^	—	—
MEF_75_ [l/s]	7.25 ± 1.92	7.08 ± 1.79	—	—	0.4^***^	—	—
MEF_75_ [%]	96.14 ± 16.06	94.14 ± 16.12	—	—	0.5^***^	—	—
**T-Group n = 28**							
FVC [l]	4.73 ± 0.91	4.68 ± 0.86	4.70 ± 0.83	0.2	0.3	0.05	0.8
FVC [%]	104.89 ± 11.80	104.00 ± 9.95	105.10 ± 10.5	0.1	0.4	0.05	0.7
FEV_1_[l]	3.95 ± 0.77	3.86 ± 0.74	3.86 ± 0.71	0.2	0.1	0.07	0.6
FEV_1_[%]	102.63 ± 13.61	99.89 ± 12.16	100.81 ± 12.48	0.1	0.07	0.07	0.5
FEV_1_/FVC [%]	83.98 ± 7.55	82.76 ± 7.86	82.40 ± 7.92	0.3	0.2	0.9	0.2
PEF [l/s]	7.66 ± 1.69	7.41 ± 1.98	7.61 ± 2.21	0.2	0.1	0.01	0.9
PEF [%]	89.22 ± 13.50	85.48 ± 13.13	88.23 ± 15.98	0.2	0.07	0.01	0.9
MEF_25_ [l/s]	2.27 ± 0.77	2.16 ± 0.73	2.19 ± 0.76	0.2	0.1	0.5	0.3
MEF_25_ [%]	93.40 ± 29.63	89.07 ± 29.19	91.00 ± 31.75	0.3	0.1	0.5	0.4
MEF_25–75_ [l/s]	4.27 ± 1.24	4.04 ± 1.20	4.01 ± 1.18	0.2	0.02	0.3	0.2
MEF_25–75_ [%]	93.30 ± 24.96	87.89 ± 23.33	87.88 ± 24.02	0.2	0.02	0.2	0.2
MEF_75_ [l/s]	6.82 ± 1.76	6.58 ± 1.76	6.57 ± 1.88	0.03	0.08	0.1	0.6
MEF_75_ [%]	92.37 ± 18.42	88.63 ± 16.27	89.19 ± 18.88	0.03	0.07	0.1	0.4
**E-Group n = 28**							
FVC [l]	5.03 ± 1.15	4.97 ± 1.14	5.04 ± 1.22	0.7	0.3	0.08	0.7
FVC [%]	103.30 ± 14.78	102.30 ± 16.44	103.59 ± 16.64	0.5	0.4	0.1	0.6
FEV_1_[l]	4.15 ± 1.07	4.13 ± 0.99	4.19 ± 1.04	0.2	0.1	0.2	0.6
FEV_1_[%]	100.63 ± 19.66	99.85 ± 17.90	101.22 ± 19.42	0.2	0.2	0.2	0.5
FEV_1_/FVC [%]	82.82 ± 11.37	83.09 ± 7.61	83.10 ± 7.95	0.4	0.3	0.7	0.4
PEF [l/s]	8.07 ± 2.67	7.76 ± 2.16	7.66 ± 2.37	0.5	0.1	0.8	0.1
PEF [%]	87.52 ± 24.12	84.30 ± 18.84	83.22 ± 22.31	0.5	0.15	0.8	0.1
MEF_25_ [l/s]	2.46 ± 0.86	2.40 ± 0.91	2.50 ± 0.88	0.5	0.2	0.4	0.6
MEF_25_ [%]	97.11 ± 33.30	94.07 ± 34.53	98.44 ± 34.68	0.4	0.2	0.4	0.6
MEF_25–75_ [l/s]	4.44 ± 1.34	4.32 ± 1.26	4.41 ± 1.30	0.7	0.4	0.2	0.9
MEF_25–75_ [%]	92.70 ± 26.20	90.07 ± 24.77	91.85 ± 25.81	0.6	0.3	0.2	0.8
MEF_75_ [l/s]	7.21 ± 2.45	6.99 ± 2.10	6.91 ± 2.19	0.2	0.09	0.7	0.3
MEF_75_ [%]	91.52 ± 27.22	88.89 ± 23.38	87.89 ± 24.61	0.2	0.1	0.7	0.3
**T/E-Group n = 28**							
FVC [l]	5.32 ± 0.95	5.35 ± 0.95	5.25 ± 0.94	0.4	0.9	0.2	0.2
FVC [%]	106.90 ± 7.87	107.5 ± 11.71	105.52 ± 9.00	0.5	0.8	0.3	0.2
FEV_1_[l]	4.35 ± 0.67	4.35 ± 0.68	4.33 ± 0.68	0.8	0.7	0.7	0.5
FEV_1_[%]	103.10 ± 8.22	103.44 ± 10.13	102.81 ± 8.25	0.6	0.9	0.2	0.5
FEV_1_/FVC [%]	82.00 ± 5.71	81.91 ± 5.45	82.53 ± 4.79	0.06	0.9	0.05	0.08
PEF [l/s]	8.22 ± 1.93	7.68 ± 1.65	7.99 ± 1.64	0.03	0.003	0.3	0.2
PEF [%]	87.81 ± 16.63	82.04 ± 13.61	85.15 ± 12.61	0.02	0.002	0.2	0.2
MEF_25_ [l/s]	2.22 ± 0.55	2.35 ± 0.61	2.24 ± 0.49	0.6	0.15	0.3	0.9
MEF_25_ [%]	86.22 ± 21.87	91.37 ± 23.20	87.41 ± 19.78	0.6	0.14	0.4	0.8
MEF_25–75_ [l/s]	4.39 ± 0.78	4.38 ± 0.80	4.43 ± 0.71	0.5	0.6	0.3	0.4
MEF_25–75_ [%]	90.67 ± 15.06	90.30 ± 14.40	91.59 ± 12.89	0.3	0.5	0.3	0.4
MEF_75_ [l/s]	7.44 ± 1.66	6.99 ± 1.60	7.32 ± 1.34	0.01	0.005	0.1	0.3
MEF_75_ [%]	92.96 ± 17.39	87.26 ± 13.63	91.26 ± 12.42	0.02	0.005	0.1	0.3

Legend: SD - standard deviation; min-max - minimum and maximum value range; I, II, III - order of measurements; *result of Friedman ANOVA; **Post-hoc Dunn’s test; ***Wilcoxon signed-ranked test.

### Absolute differences

Acute respiratory effects after exposure to t-cigarette, e-cigarette (E-Group and T/E-Group) or after simulation of e-cigarette use are presented in Tables 2 and 3.

Among T-Group, a reduction in O_2_ saturation was observed immediately after exposure (minute 1) with a return to the baseline values after 30 minutes from cigarette smoking. The simulation of the use of an e-cigarette as well as the active use of an e-cigarette did not significantly affect the value of O_2_ saturation obtained during measurements. The active use of a traditional cigarette or e-cigarette was associated with a significant decrease (p = 0.0001) in exhaled FeNO values observed in the first minute after exposure: mean by 2.1 ppb in T-Group, by 1.5 ppb in E-Group and by 2.2 ppb in T/E-Group. At 30 min after exposure, FeNO values increased, reaching a level comparable (T-Group, T/E-Group) or higher (mean by 1 ppb, E-Group) to the baseline values (Table 2).

Among T-Group, there was a significant increase in exhaled CO values observed in the first minute after exposure (mean by 3.67 ppm, p = 0.0001) which remained elevated after 30 minutes from exposure (mean by 2.67 compared to baseline values). Among dual users, CO values observed after 30 min of e-cigarette use were decreased by 0.97 ppm compared to the initial values (Table 2).

Active cigarette smoking or e-cigarette use also significantly affected the temperature of exhaled air. Statistically significant increases in exhaled air temperature was observed at minute 1 and minute 30 after e-cigarette use in E-Group and dual users. Cigarette smoking was associated with an increase in exhaled air temperature at 30 minutes following exposure. Among cigarette smokers (T-Group) and dual users (T/E-Group), significant decreases in peak expiratory flow (PEF) and maximal expiratory flow at 75% of FVC (MEF_75_) were observed at the first minute after cigarette or e-cigarette use (Table 3). At 30 min following exposure, the values of spirometric variables were similar to those observed at the baseline measurement (Table 3).

There were no statistically significant changes in measured lung function parameters after simulation of e-cigarette use in the control group.

### Relative differences

Following exposure and compared with Control subjects, E-Group, T-Group and T/E-Group had statistically significant decreases (p < 0.05) in MEF_25_. None of the spirometric values differed statistically at minute 30 compared to baseline values. There was a decrease in O_2_ saturation in T-Group at minute 1 and a return to baseline values at minute 30. Compared with baseline values, FeNO levels were significantly (p = 0.0001) lower in E-Group, T-Group and T/E-Group at minute 1 with a return to baseline values observed at minute 30. CO levels in exhaled air increased directly after exposure only in T-Group, and stayed increased 30 minutes after exposure. Tables 4 and 5 show the mean relative differences in the examined indices assessed at minute 1 (Table 4) and t minute 30 (Table 5) in the study groups.

**Table 4:** Mean relative differences in the examined indices assessed on minute 1.

Variable	Relative difference (%): Baseline Measurement vs Measurement on Minute 1				P value
	E-Group mean ± SD	T-Group mean ± SD	T/E-Group mean ± SD	C-Group mean ± SD	
FVC [l]	1.0 ± 4.1	1.5 ± 4.9	−0.5 ± 6.8	−0.8 ± 3.0	0.2
FEV_1_[l]	2.3 ± 5.7	2.8 ± 7.2	−0.2 ± 6.4	−0.3 ± 3.7	0.4
FEV_1_/FVC [%]	1.3 ± 4.3	1.3 ± 5.9	0.2 ± 2.7	0.6 ± 2.4	0.8
PEF [l/s]	3.8 ± 12.0	4.6 ± 13.5	5.5 ± 9.9	2.4 ± 13.0	0.9
MEF_25_ [l/s]	5.3 ± 16.0	4.3 ± 14.4	−7.3 ± 19.1	3.5 ± 15.6	0.02
MEF_25–75_ [l/s]	4.2 ± 11.8	4.8 ± 12.5	−0.5 ± 11.1	0.9 ± 9.6	0.7
MEF_75_ [l/s]	3.1 ± 10.5	3.0 ± 15.7	4.9 ± 10.7	1.3 ± 13.6	0.6
FeNO [ppb]	7.3 ± 13.4	13.1 ± 11.2	12.8 ± 16.7	0.3 ± 13.4	0.0002
O2 Saturation [%]	−0.1 ± 1.1	0.6 ± 1.1	0.2 ± 0.8	0.2 ± 0.7	0.09
Exhaled air temperature [°C]	−0.5 ± 1.2	0.0 ± 1.1	−0.5 ± 0.9	−0.2 ± 1.1	0.4
Exhaled CO level [ppm]	−11.9 ± 27.7	−154.4 ± 115.1	−1.1 ± 13.8	−11.1 ± 31.4	0.0001

Legend: SD - standard deviation; p - result of Kruskal-Wallis test.

**Table 5:** Mean relative differences in the examined indices assessed on minute 30.

Variable	Relative difference (%): Baseline Measurement vs Measurement on Minute 30				P value
	E-Group mean ± SD	T-Group mean ± SD	T/E-Group mean ± SD	C-Group mean ± SD	
FVC [l]	−0.2 ± 3.9	0.2 ± 5.4	1.4 ± 4.4	—	0.4
FEV_1_[l]	1.0 ± 6.3	1.7 ± 7.3	0.4 ± 5.2	—	0.8
FEV_1_/FVC [%]	1.3 ± 4.4	1.6 ± 5.2	−1.0 ± 3.8	—	0.09
PEF [l/s]	5.5 ± 15.3	0.2 ± 17.0	1.0 ± 17.0	—	0.5
MEF_25_ [l/s]	0.8 ± 19.3	1.4 ± 13.3	−2.8 ± 16.1	—	0.6
MEF_25–75_ [l/s]	2.7 ± 11.2	3.8 ± 13.2	−2.0 ± 10.6	—	0.5
MEF_75_ [l/s]	4.1 ± 14.6	1.8 ± 16.4	−0.2 ± 15.0	—	0.9
FeNO [ppb]	−8.4 ± 18.6	−3.9 ± 11.9	−5.6 ± 18.5	—	0.5
O_2_ Saturation [%]	−0.1 ± 0.9	−0.0 ± 1.1	0.1 ± 1.0	—	0.6
Exhaled air temperature [°C]	−0.7 ± 1.3	−0.9 ± 1.0	−0.6 ± 1.0	—	0.4
Exhaled CO level [ppm]	−8.9 ± 26.9	−117.6 ± 90.5	11.0 ± 19.2	—	0.0001

Legend: SD - standard deviation; p - result of Kruskal-Wallis test.

### Multivariate analysis

Relative differences in variables measured at baseline and minute 1 then separately between baseline and minute 30 were compared pairwise between E-Groupand T-Group, and between E-Group and T/E-Group.

A comparison of the relative differences obtained in E-Group with those obtained in T-Group showed differences in terms of O_2_ saturation (baseline-first point: p = 0.01), CO level (baseline-first point: p < 0.001; baseline-second point: p = 0.001), and PEF (baseline-second point: p = 0.05).

A comparison of relative differences obtained in E-Group with those obtained in T/E-Group showed differences in terms of FeNO (baseline-first point: p = 0.06), CO level (baseline-second point: p = 0.009), FEV_1_ (baseline-first point: p = 0.03), FEV_1_/FVC (baseline-second point: p = 0.08), MEF_25–75_ (baseline-first point: p = 0.04, baseline-second point: p = 0.06) and MEF_25_ (baseline-first point: p = 0.0007).

According to the results of the multivariate analysis, sex has a statistically significant impact on CO level on minute 1 (p = 0.04) and on minute 30 (p = 0.05) in T-Group vs E-Group and in case of CO level on minute 1 (p = 0.06) in a T-Group vs C-Group. In case of O_2_ saturation in T/E-Group vs C-Group there were effects of sex (p = 0.03) and height (p = 0.02) and in case of O_2_ saturation in a group of E-Group vs C-Group there was a marginally significant effect of sex (p = 0.09).

## Discussion

The use of electronic cigarettes is portrayed and advertised in popular mass media as a safer alternative to traditional tobacco smoking. However, the evidence regarding this matter is not unequivocal^21–24^. A report prepared by Public Health England repeatedly emphasized that the use of e-cigarettes is up to 95% less harmful in comparison with tobacco cigarettes smoking^21^. The opposite position is presented by the European Respiratory Society and American Heart Association^22,23^. Both societies indicate that there is no conclusive evidence on the impact of e-cigarettes on health^22,23^. Furthermore, the World Health Organization indicates that the effectiveness of using e-cigarettes as a tool to help quit or reduce tobacco smoking is questionable^24^.

Our study is one of the largest investigations aimed at acute health effects in response to e-cigarette use and the first conducted in a “real-life” scenario including subjects who regularly use e-cigarettes. Following exposure, statistically significant decreases in PEF and MEF_75_ were found in T-Group and T/E-Group compared with Control Subjects. Five minutes of e-cigarette use was sufficient to trigger a decrease in fractional exhaled nitric oxide (FeNO) levels both in exclusive e-smokers and dual users. Active e-cigarette use or cigarette smoking also evoked significant increases in the temperature of exhaled air. Moreover, reduced baseline FeNO values were measured after cigarette (T-Group) or e-cigarette (E-Group and T/E-Group) use.

The influence of tobacco smoking on lung function has been widely examined^25–27^. In general long-term tobacco smokers have decreased values of forced vital capacity (FVC), forced expiratory volume in 1 second (FEV_1_), Tiffeneau index and forced expiratory flow at 25–75% FVC [FEF_25–75_]^26^. In our study, no difference in airflow at baseline was observed, which could be explained by a short history of exposure to tobacco smoking. In young smokers, changes in spirometric parameters are not as severe as among adult, long-term smokers^25^.

In our study, exposure-related decreases in MEF_75_ and PEF measured at minute 1 occurred in cigarette smokers (T-Group) and dual users (T/E- Group). Vardavas *et al*. did not observe any significant changes in the spirometric results at 5 minutes after using an e-cigarette^10^. Flouris *et al*. also did not observe changes in FEV_1_/FVC values immediately after using an e-cigarette^12^. In the same study, smoking a tobacco cigarette was associated with a mean decrease of 7.2% in FEV_1_/FVC. Similar effects were found by Chorti *et al*. who showed a decrease in FEV_1_/FVC immediately after smoking a tobacco cigarette^28^.

Some reports address the role of FeNO measurement in the assessment of respiratory response to smoking^29,30^. Baseline measurements show lower levels of FeNO in tobacco smokers than in non-smokers with the difference reaching 6–9 ppb^30,31^. The effect depends on the daily number of smoked cigarettes^31^. However, analogous evidence regarding e-cigarette using, obtained in a cross-sectional manner, is not available. In our study, all three smoking groups (T-Group, T/E-Group and E-Group) had lower FeNO levels on baseline measurement compared to the control group. This observation may indicate potential long-term effects and health consequences of e-cigarette use. Such a finding should not be neglected because reduction of endogenous nitric oxide synthesis may increase the risk of hypertension, atherosclerosis, peripheral vascular disease and increased incidence of respiratory tract infections^32–35^.

FeNO measurement has been used to explore acute responses to e-cigarette using and the reports include various results^10,11,33^. Tzortzi *et al*. showed that a 30-minute passive exposure to e-cigarettes resulted in significantly decreased FeNO levels (from 24.16 ppb at baseline to 22.35 ppb after exposure) among 40 healthy nonsmokers^36^. Vardavas *et al*. showed that using an e-cigarette for 5 minutes was associated with a 16% decrease in FeNO level, in a group of 30 subjects^10^. A similar observation was reported by Lappas *et al*.^37^. After 5 minutes of e-cigarette use (e-liquid containing 12 mg/ml nicotine), a significant decrease in FeNO levels were observed among healthy adults (n = 27) and subjects with intermittent asthma (n = 27)^37^. The opposite results were obtained by Schober *et al*. in 9 adult tobacco smokers who were asked to smoke an e-cigarette. This experiment showed a post-exposure increase in FeNO levels, in 7 out of 9 study subjects^11^. Moreover using nicotine-free e-cigarettes was not associated with cross-exposure changes in FeNO levels^11,33^. Palamidas *et al*. also did not observe statistically significant changes in FeNO levels after 10 min of e-cigarette use^38^. The results of our study are in line with observations obtained by Vardavas *et al*.^10^ and Lappas *et al*.^37^. A statistically significant decrease in FeNO levels was seen immediately after smoking a cigarette and using e-cigarette.

Nitric oxide is a gaseous mediator with play a significant role in a number of biological processes^39^. Nitric oxide is also a sensitive marker, correlated with eosinophilic inflammation and oxidative stress in the airways^10,39^. Changing the nitric oxide content in the exhaled air immediately after using the e-cigarette may suggest that the aerosol from the e-cigarette disturbs the pulmonary homeostasis, perhaps in the form of inflammation in response to irritation by the e-cigarette aerosol. Lack of changes in FeNO levels in studies by Ferrari *et al*.^33^ and Schober *et al*.^11^, when using nicotine-free e-cigarettes may indicate that nicotine was the main factor responsible for the change in FeNO levels and differences between our study and previously reported studies.

The studies available in the literature indicate that cigarette smokers have lower oxygen saturation values compared to non-smokers^40^. In this study, immediately after cigarette smoking, the saturation value decreased on average by 0.6%. Both active use of the e-cigarette and simulation of use did not affect the saturation values observed before and after the exposure. This effect can be caused by carbon monoxide emitted during cigarette smoking.

Supporters of e-cigarette use base their attitude on the fact that e-cigarette use is free from the combustion process and is not associated with inhalation of carbon monoxide. In our experiment no significant changes in CO concentration in exhaled air were observed but both E-Group and T/E-Group had higher baseline values of CO in comparison with control subjects. Similar findings were obtained by Schober *et al*.^11^, Vansickel *et al*.^15^, and Chorti *et al*.^28^.

Palamidas *et al*. showed no statistically significant changes in exhaled air temperature after 10 min of ad lib e-cigarette use^38^. In our study, analysis of direct differences indicates, that five minutes of e-cigarette or t-cigarette use was sufficient to increase exhaled breath temperature. However, among E-Group and T/E-Group, exhaled breath temperature increased immediately after the exposure, while in T-Group the increase was observed only after 30 min of exposure. Measurement of exhaled breath temperature is a relatively new method used to detect and monitor pathological processes in the human respiratory system^41^. One of the factors causing an increase in exhaled air temperature is inflammatory process^41,42^. Glynos *et al*. revealed that exposure to e-cigarette vapor can trigger inflammatory responses and alters respiratory system mechanics in mice, and this effect is particularly visible when using flavored e-liquid with nicotine^43^. Kralimarkova *et al*. suggested that cigarette smoke may increase the temperature of exhaled air as a result of increased blood flow in the blood vessels of the respiratory tract and the probability of inflammation development^20,42^. It cannot be ruled out that a similar mechanism may take place during e-cigarette use, as an airways response to irritation by the e-cigarette aerosol. An increase in exhaled breath temperature may result from inflammatory process, caused by nicotine or flavoring chemicals in e-cigarette (especially diacetyl or 2,3-pentanedione, which are commonly used to add flavors to e-liquids)^44^.To our best knowledge, this is the first study reporting the increase in exhaled air temperature after the use of an e-cigarette.

E-cigarette using involves exposure to a number of substances including propylene glycol, glycerin and nicotine. There is also a wide spectrum of chemicals that are responsible for a multitude of flavors contained in e-liquid. Currently, the precise determinations of individual exposure to chemicals as well as long-term health consequences of e-cigarette using are unknown.

Our study addressed the acute respiratory effects of e-cigarette using. The principal findings include small but statistically significant changes in airflow and in FeNO levels. Moreover the results of multivariate analyses suggest that in terms of immediate respiratory responses (spirometric variables, FeNO, temperature) to applied exposure E-Group do not differ from T-Group. This finding deserves more attention in future investigations.

According to the results of the multivariate analysis, sex and height are only associated with O_2_ saturation and CO levels in selected groups. We did not highlight these effects in the manuscript because these two parameters were not crucial in our study (no impact of e-cigarette on CO levels is well known; changes in O_2_ saturation only in smokers, without significant clinical relevance). In spite of the longer smoking history among dual users compared to conventional tobacco smokers, the results of multivariate analysis revealed that there were no impact of prior smoking history on the observed changes after exposure to a cigarette or e-cigarette.

Our study is not free from limitations. First, our study was focused only on the assessment of short-term, immediate respiratory effects of smoking an e-cigarette. Subjects used their own e-cigarette freely, and the number and time of puffs were not controlled by the test protocol. It cannot be excluded that between-subject differences in device used in the experiment might have affected the group results. Another potential limitation is the small number of subjects in each study group. Similarly as Ferrari *et al*.^33^, minimum sample size was based on a sophisticated calculation involving previously reported data^10,11,45^, so the sample size (n = 30/each group) included in our study, allows for inference based on the presented differences in respiratory responses to cigarette and e-cigarette. However some published reports are based on similar study sizes thus our results can be compared to the results available in the literature. Another important point is that our study seems to be one of the largest experimental studies in the field of e-cigarette using that follows a “real-life scenario”, including subjects, who regularly use e-cigarettes. Moreover, an important point in our study protocol is that we made a distinction between exclusive e-cigarette users and dual users, and the results pertinent to e-cigarette using can be directly compared to those pertinent to cigarette smoking. However it would also be of interest to study the respiratory effects of using e-cigarettes by pure tobacco smokers.

## Conclusions

Acute, short term respiratory responses to the use of e-cigarettes include a decrease in concentration of NO in exhaled air (FeNO), increase in airway temperature and decrease in airflow (PEF, MEF_25_, MEF_75_) in tobacco smokers and dual users (tobacco smokers and e-cigarette users). The pattern of respiratory responses to the use of an e-cigarette by e-cigarette users is similar to the responses to smoking a tobacco cigarette by tobacco smokers.

## Data Availability

The datasets generated during and analysed during the current study are available from the corresponding author on reasonable request.
